# Dendritic cell density and activation status in human breast cancer – CD1a, CMRF-44, CMRF-56 and CD-83 expression

**DOI:** 10.1038/sj.bjc.6600132

**Published:** 2002-02-12

**Authors:** B J Coventry, P-L Lee, D Gibbs, D N J Hart

**Affiliations:** Department of Surgery, University of Adelaide, Royal Adelaide Hospital, North Terrace, Adelaide, South Australia 5000, Australia; Haematology/Immunology Research Group, University of Otago, Canterbury Health Laboratories, PO Box 151, Christchurch, New Zealand; Mater Medical Research Institute, Mater Hospital, Brisbane, Queensland 4101, Australia

**Keywords:** dendritic cells, breast carcinoma, CD1a, CMRF-44, CMRF-56, CD83, DC activation

## Abstract

Low CD1a-positive putative dendritic cell numbers in human breast cancer has recently been described and may explain the apparent ‘poor immunogenicity’ previously reported in breast cancer. Little attention has been given to dendritic cell activation within the tumour microenvironment, which is another reason why the *in-situ* immune response may be severely deficient. We have therefore examined CD1a expression as a marker for dendritic cells, together with CMRF-44 and -56 as markers of dendritic cell activation status, in 40 human breast cancers. The results demonstrate few or no CD1a-positive putative dendritic cells and minimal or no expression of the dendritic cell activation markers. Both dendritic cell number and dendritic cell activation appear substantially deficient in human breast cancers, regardless of tumour histological grade.

*British Journal of Cancer* (2002) **86**, 546–551. DOI: 10.1038/sj/bjc/6600132 www.bjcancer.com

© 2002 Cancer Research UK

## 

Breast adenocarcinoma has traditionally been thought of as a ‘non-immunogenic’ tumour, despite numerous observations concerning the notable density of the T-cell infiltrate in human and animal breast cancers (Zhang *et al*, 1998). However, specific anti-tumour responses by autologous T-lymphocytes infiltrating human breast tumours have been documented using short-term cultured breast carcinoma cells *in-vitro* (Baxevanis *et al*, 1994), and more widespread interest in the possibility of generating immunotherapeutic responses against breast cancer has developed. Dendritic cells (DC) have been identified as the specialist leukocyte population capable of initiating and directing immune responses. Studies of gastric, thyroid, lung and colorectal carcinomas have shown that the density of putative dendritic cells (DC) (CD1a; S-100 positive) is a predictor of survival (Tsujitani *et al*, 1987, 1988, 1990, 1992; Ambe *et al*, 1989; Schroder *et al*, 1988; Zeid and Muller, 1993). Equally, more recent studies suggest minimal recruitment and activation of DC in renal, prostate and bladder cancers (Troy *et al*, 1998a,b, 1999) and that S-100 staining may correlate with an activated DC subset (Troy *et al*, 1998a). Moreover, it has been shown that there is an inverse correlation between tumour differentiation grade and the DC infiltrate for some tumour types (Becker, 1992). Recent studies have demonstrated a low number of CD1a positive cells in human breast carcinomas (Hillenbrand *et al*, 1999) and these have been able to be extracted using specific dissaggregation techniques (Coventry *et al*, 1997). CD1a molecules appear to be expressed on epithelial associated DC during the antigen capture and processing phase, reducing in density as the DC phenotype develops full capacity for antigen presentation *in-vivo*. The possibility that the ‘non-immunogenicity’ of breast cancers may, in part, be due to low DC infiltration, loss of DC from the tumour microenvironment or lack of DC activation, requires further investigation.

DC are derived from bone marrow precursors and recent data suggests that there are epithelial and non-epithelial associated subsets, distinguishable on the basis of CD1a expression. Surveillance DC require defined ‘danger’ signals derived from microbial organisms triggered by the innate immune response (perhaps lacking in some cancers) to initiate antigen uptake, migration and differentiation/ activation of DC co-stimulatory activity. Further control of DC co-stimulatory activity occurs as a result of the DC/T-cell interaction (Hart, 1997). Acquisition of CD86 is an important end point of DC differentiation, signifying full co-stimulatory capacity (Thomas and Lipsky, 1996; Hart, 1997; McLellan *et al*, 1999). Recent evidence suggests other mechanisms may downregulate DC activation, depending on DC maturity (McLellan *et al*, 2000)

Effective antigen presentation within the tumour microenvironment for specific anti-tumour immune responses might be associated with differentiated, activated DCs. Equally it is possible that tumours may fail to recruit DC or activate DC thereby evading an effective anti-tumour response. CMRF-44 monoclonal antibody (mAb) acts as a marker of allostimulatory activity in cultured peripheral blood mononuclear cells (Hock *et al*, 1994; Vuckovic *et al*, 1998), and defines an early differentiation activation marker on DC present on only a small number of peripheral tissue DC. Likewise the mAb CMRF-56 reacts with another early activation antigen expressed in high density on DCs, but not with other resting or activated leukocytes (Hock *et al*, 1999). Both of these markers show a strong correlation with a third DC differentiation/ activation antigen CD83 (Zhou and Tedder, 1995) as well as the up-regulation of cells and other cytoplasmic markers. Interleukin 10 (IL10) expression as a potential inhibitor of T-cells and DC is considered and discussed.

This study investigates the expression of the epithelial DC marker CD1a with that of CMRF-44, CMRF-56 and CD83 to determine the activation state of DC in human breast cancer.

## MATERIALS AND METHODS

The studies were performed in two collaborating laboratories and are reported in two parts – Part I (Adelaide) and Part II (Christchurch). The studies were approved by and met the ethical standards of both institutions.

### Tissues and processing

#### Part I

Fresh tissue was frozen in liquid nitrogen from samples taken at routine surgery from 30 infiltrating ductal breast carcinomas (IDC) (IDC grade I =10, IDC grade II =10, IDC grade III =10), then stored at −80°C for later use. Samples were cut from intratumoural portions of tumours that were palpable (rather than mammographically detected). Four to five μm serial sections (Leica Cryostat) were mounted on gelatinized slides, left overnight, fixed in cold (4°C) acetone for 10 min and air dried for a minimum of 2 h.

All sections were pre-blocked with 10% goat or normal horse serum in phosphate-buffered saline (PBS) for 20 min.

#### Part II

Ten cases of breast cancer were obtained, frozen in liquid nitrogen and processed similarly.

### Immunohistochemical techniques

#### Monoclonal antibodies Part I

Mouse monoclonal anti-human antibodies, diluted in 10% normal horse serum (NHS), were used at the following concentrations, CD1a at 1/500 (Dako, Denmark), CMRF 44 and CMRF 56 both at 1/50 (D Hart, New Zealand), mouse isotype negative control 2 μg ml^−1^ (Zymed, CA, USA). The anti-interleukin 10 antibody 12G8 (IgG; Schering, NJ, USA) was used at 1/500 dilution in separate studies briefly mentioned here. Biotinylated rabbit anti-mouse IgG/IgM second antibody at 1/500 (Dako, Denmark), and streptavidin-HRP at 1/1000 (Pierce, USA). Single-staining protocol reactions were carried out at room temperature. PBS ×3 rinses were used after each staining step. Following blocking with 10% NHS, sections were incubated for 1 h with the primary antibody, (CD1a for 1 h, or CMRF 44/56 overnight).

#### Part II

The anti-CD3 (OKT3, IgG_2a_), antibody was produced from a hybridoma obtained from the American Type Culture Collection (ATCC, Rockville, MD, USA). The antibodies CMRF-12 (CD45, IgG_1_), CMRF-15 (negative control, IgM), CMRF-31 (CD14, IgG_2a_), CMRF-56 (activation antigen, IgG_1_) and CMRF-44 (activation antigen, IgM) were produced and characterized in this (DNJH) laboratory. The anti-CD83 mAb was purchased from Immunotech. The anti CD16 mAb (HuNK-2, IgG_2a_) was a gift from Professor IFC McKenzie (Melbourne, Australia). The CD1a mAb (Na1/34, IgG_2a_), was a gift from Dr A McMichael (UK). The negative control mAb Sal5 (IgG_2a_) and X63 (IgG_1_), were gifts from Professor H Zola (Adelaide, Australia). Phycoerythrin-conjugated anti-CD45 and HLA-DR were purchased from Becton Dickinson (Australia). PE-conjugated anti-CD83 was purchased from Immunotech.

### Immunoperoxidase labelling

#### Part I

Biotinylated secondary antibody for 1 h. Endogenous peroxide blocking (PBS and 0.5% H_2_O_2_ for 20 min) was followed by a 1 h incubation in SHRP. Sections were reacted with nickel chloride enhanced diaminobenzidene (DAB), and counterstained with methyl green (Coventry *et al*, 1994, 1995) for higher detection sensitivity. Lymph node sections from melanoma were used as positive controls.

#### Part II

Specimens were cut, fixed and blocked as above. After incubation with the primary antibody, slides were washed three times in PBS and peroxidase-conjugated goat anti-mouse antibody (PGAM) (DAKO) applied. The slides were washed in tris buffered saline (TBS) then DAB applied. The reaction was terminated after 10–15 min by washing in PBS. Slides were counterstained, fixed and mounted as above.

### Immunofluorescence labelling

#### Part II only

Specimens were cut, fixed and blocked as above. The primary antibody mix was applied for 30 min at room temperature, then the slides were washed three times in PBS. Fluorescein isothyanate-conjugated sheep anti-mouse antibody (Silenus) was applied for 30 min. After washing three times in PBS, the slides were blocked with 10% mouse serum for 10 min. A Phycoerythrin-conjugated secondary antibody was applied for 30 min. After washing three times in PBS, the slides were mounted in glycerol-gelatin with DABCO as an anti-fade agent. Slides were stored in darkness at −20°C and examined within 24 h of preparation.

### Characterization of cellular infiltrate

#### Part I

CD1a, CMRF 44 and 56 density assessment was performed by counting 50 random fields per frozen section, or as many fields as possible if the section was smaller. The mean of each section represented the density of the infiltrate (field size 0.375 mm diameter; 0.44 mm^−2^; 400× magnification). This allowed estimation of the density and distribution of different cell types in the infiltrate compared with CD1a^+^ staining to be expressed as cells per mm^−2^ (i.e. as in Part II).

#### Part II

DC were identified in single label immunohistological studies by size, morphology and staining with the appropriate marker. The cells were counted in at least 10 medium power fields using a calibrated graticule, then the number of cells per mm^−2^ was calculated. DC were identified in double label immunofluorescence studies as CD45^+^ (i.e. Phycoerythrin labelled) cells that were negative (i.e. FITC negative) for the lineage markers CD3, CD14, CD16 and CD19.

## RESULTS

### Identification of putative dendritic cells

#### Part I

CD1a^+^ cells were present in 50% of the breast tissue samples (*n*=30). The density of the infiltrate was variable between samples (range 0.00–6.05/HPF; 0.00–13.75 mm^−2^; mean=2.49 cells mm^−2^).

There was no association between density of CD1a^+^ cells and tumour grade (Kruskal-Wallis, *P*>0.05).

DC occurred in small numbers relative to the number of CD3^+^ cells. CD1a^+^ cells, probably epithelial DC, were present in the greatest numbers, as previously reported (Hillenbrand *et al*, 1999). The CD1a^+^ cells tended to occur in loose clusters (distinct from lymphoid aggregates), so obtaining a count that accurately reflected their numbers was difficult. In some instances CD1a staining was thought to be present on a sub-population of macrophages.

**Figure 1:**
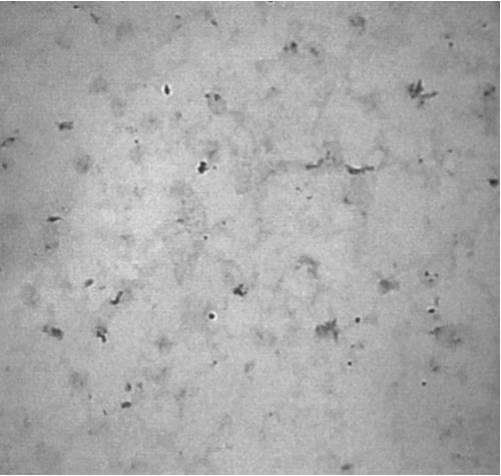
CMRF-44 staining in human breast cancer (Part 1) (×200).

**Figure 2:**
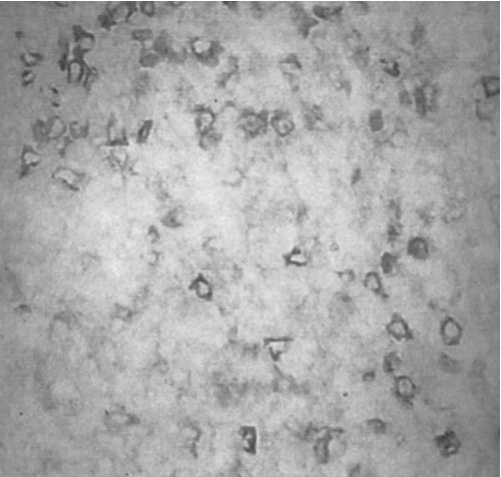
CMRF-56 positive cells in human breast cancer (Part 1) (×200).

**Figure 3:**
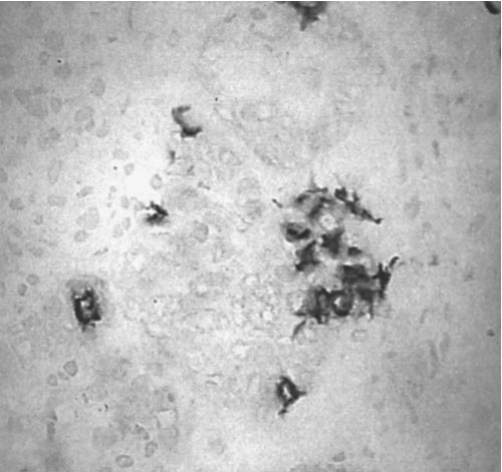
CD1a positive cells in human breast cancer (Part 1) (×200).

Faint CMRF-44^+^ staining was observed in only one of the 30 breast carcinoma sections examined in this study ( Figure 1

), with the same sample demonstrating relatively strong CMRF-56^+^ immunostaining ( Figure 2

), with CD1a^+^ cells found clustered around a tumour island ( Figure 3

). CD1a, CMRF-44 and -56 staining in normal breast tissues was sparse or absent.

#### Part II

The immunoenzymatic staining identified a similar number of CD1a^+^ DC in the second study (*n*=10), with a similar range (0–16.6 mm^−2^). The mean number of CD1a^+^ cells was 5.5 mm^−2^ and CD3 T-lymphocytes was 114.5 mm^−2^. Again limited staining with the activation markers was noted (CMRF 44 >CD83 >CMRF-56). There was a trend for the activated DC to be associated with increased T-lymphocyte numbers (see below). The double label immunofluorescence studies identified very similar numbers of Lin^−^/CD45^+^ DC, most of which were CD1a^+^, but increased CD1a staining on macrophages was evident in some cases. Counts were hampered by high non-specific background staining as a consequence of auto-fluorescence. Nonetheless, the CD1a counts obtained by this method were comparable with those obtained by immunohistochemistry. Comparing the lineage-negative cell counts and the CD1a^+^ cell counts suggests that a subset of DC in breast cancers express the epithelial DC marker CD1a ( Table 1

**Table 1:**
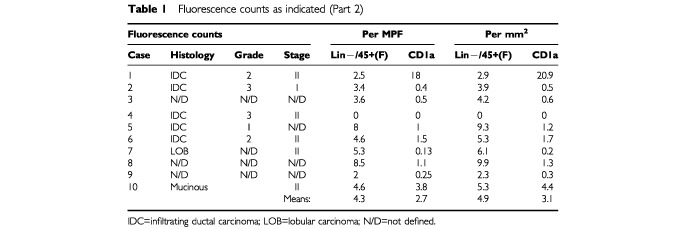
Fluorescence counts as indicated (Part 2)

).

### Identification of activated dendritic cells

Although only one of 30 specimens was positive for CMRF-44 in Part I using DAB enhanced immunoenzymatic methods, limited numbers of CD83 and CMRF-44^+^ cells were identified in most specimens, using the alternative immunoenzymatic method or fluorescence method (Part II). CMRF-56^+^ cells were only identified in one specimen (for both Parts I and II; *n*=40) although other specimens had peri-tumoural lymphoid aggregates which contained cells that stained weakly with CMRF-56 (Table 1).

An attempt was made to assess co-expression of CD1a and CD83 using immunofluorescence techniques but high background fluorescence made identification of phycoerythrin-labelled CD83^+^ cells difficult. However, since no CD1a^+^/CD83^+^ cells were identified (as few CD83^+^ cells could be identified by indirect immunofluorescence) the conclusion that the two markers identify different populations of cells could not be established with certainty. Nonetheless, the immunofluorescence data confirmed the low number of CD83^+^ cells present.

#### Location of CD1a^+^ cells

The CD1a^+^ cells were present in the stroma between islands of tumour cells and in more differentiated tumours were situated around the ductal formations.

#### Interleukin 10 in human breast cancer

In a separate study IL10 expression was detected in 40 of 52 breast cancer specimens examined using immunohistochemical methods. The histological grade of the tumour did not correlate with the IL10 expression, nor did CD3 or CD68 expression. The IL10 staining intensity was low in all tumours and these cells were within the stroma around the ducts in well to moderately differentiated tissues (Neville, 1994).

## DISCUSSION

The finding of paucity of CD1a^+^ cells in many of the breast carcinomas examined indicated that DC mediated co-stimulation of T-lymphocytes in many breast cancers is likely to be severely deficient. It was also possible that CD1a negative DC may have been present and active within the tumour microenvironment. We excluded these possibilities by using lin- CD45^+^ staining for DC. To further investigate the DC present the CMRF-44, CMRF-56 and CD83 antibodies were used as another means of detecting DC and to assess their activation state. These studies showed that few mature or activated DC could be detected within breast tumours, implying that DC numbers and activation were indeed absent or very low in all but one tumour in the 30 tested.

It has been proposed that CD1a acts as a lipid/carbohydrate carrying glycoprotein, similar in function to major histocompatability complex (MHC) molecules for peptides, with antigen presentation functions (Hillenbrand, 1994, Hillenbrand *et al*, 1999; Coventry, 1999). Interestingly, classical MHC molecules are frequently completely or partially lost from tumour cells with transition from *in-situ* to invasive growth (Garrido *et al*, 1993; Algarra *et al*, 2000). CD1a appears to be internalized and/or downregulated as the DC matures and acquires the ability to present antigen more efficiently. The association between MHC and CD1a expression in tumours is currently unclear, but this study appears to exclude major down regulation of CD1a expression as an additional deficit.

DC are found in low numbers in other tumour types (Troy *et al*, 1998a,b, 1999) and the density of CD1a positive DC has been directly correlated with improved overall survival in many solid tumour types. The findings of the present study imply that many breast cancers not only fail to recruit DC but fail to drive DC into either the antigen capture/processing phase (CD1a^+^) or the antigen presentation/co-stimulatory phase (CMRF-44/56^+^). These latter findings suggest that the tumour environment may retard or fail to facilitate maturation/activation of DC. Interleukin 10 has been shown to exert an inhibitory role over T-lymphocyte reactions and as such has been suggested as a potential inhibitory molecule affecting tumour infiltrating lymphocytes (TIL) in the breast cancer microenvironment. Although IL10 showed very low expression it may exert significant inhibition of T-cells in breast carcinoma (Neville, 1994).

DC migration may contribute to the common pathological finding of sinus histiocytosis, producing enlargement of regional, draining, non-tumour involved lymph nodes in primary breast (and other) tumours. This is a possible mechanism of induction of tumour tolerance (Steinbrink *et al*, 1999). Alternatively, mature DC may migrate or apoptose and be effectively lost from the primary tumour microenvironment (Esche *et al*, 1999; Pirtskhalaishvili *et al*, 2000). There is some evidence that anergic T-cells can induce apoptosis of DC (De Smedt *et al*, 1998; Vendetti *et al*, 2000), and that tumour cells can also cause DC apoptosis (Kiertscher *et al*, 2000).

Finally, it is unclear whether re-circulation of tumour antigen-loaded DC occurs back to the primary tumour, and whether this mechanism of T-cell activation may be defective. Vascular endothelial growth factor (VEGF) is produced by tumours to produce neo-vascularization and can function as a local and systemic inhibitor of DC differentiation, maturation and function (Gabrilovich *et al*, 1997, 1998, 1999). Active suppression of DC function has been previously noted and was observed to be HLA Class II and CD4 mediated (McLellan *et al*, 1999).

TIL isolated from human solid tumours of different types, including breast carcinoma, have demonstrated poor proliferative responses to mitogens *in-vitro* which recovers after several days co-culture with interleukin-2 (IL-2), or after culture with antigen presenting cells (APC). *In-situ* studies of TIL in breast cancers demonstrate expression of the early activation markers CD38, 43 and 69, but show little or no expression of IL-2 receptor or IL-2 protein (Coventry *et al*, 1996), suggesting anergic, tolerant T-cells. Collectively, these observations suggests that the TIL population in breast and other cancers is partially activated, but effectively rendered anergic. Tumour derived peptides loaded onto DC have been described to induce T-cell anergy (Grohmann *et al*, 1997), as one possible mechanism and again the absence of activated DC is entirely consistent with the possibility that these DC are providing tolerogenic signals. There is also evidence that anergic T-cells can inhibit antigen presenting functions of DC (Vendetti *et al*, 2000). *In-vivo* reversal of anergy using whole cell, peptide or membrane-lysate tumour vaccines which can induce tumour regression in some cases (Hersey, 1993; Soiffer *et al*, 1998; Haigh *et al*, 1999; Hart *et al*, 1999), but the mechanism remains unclear.

Nevertheless, some signs of activation are present in TIL in breast cancers which indicates that either T-cells were activated then became suppressed, or T-cell activation can by-pass the sparse, poorly activated DC within the tumour microenvironment to some minor degree. In either case, there is some optimism for the potential reversal of poor TIL activity and function using a variety of *ex-vivo* and *in-vivo* strategies to activate intra-tumoural DC, or to provide activated DC capable of effective antigen presentation to fully activate T-cells within the tumour. Recent work showing that the DC maturational type and activation differs between the intra-tumoural and peri-tumoural regions in a more advanced group of breast cancers supports our findings (Bell *et al*, 1999), and other work indicates DC maturation (and perhaps activation) can be induced by agents such as prostaglandins (Steinbrink *et al*, 2000). Taken collectively, these studies suggest that migration of DC capable of effective activation and antigen presentation into breast cancers may be defective and strategies leading to better DC migration may be required also, irrespective of how many potentially effective DC are delivered around the tumour.
